# Prediction of underlying atrial fibrillation in patients with a cryptogenic stroke: results from the NOR-FIB Study

**DOI:** 10.1007/s00415-023-11680-8

**Published:** 2023-05-10

**Authors:** B. Ratajczak-Tretel, A. Tancin Lambert, R. Al-Ani, K. Arntzen, G. K. Bakkejord, H. M. O. Bekkeseth, V. Bjerkeli, G. Eldøen, A. K. Gulsvik, B. Halvorsen, G. A. Høie, H. Ihle-Hansen, H. Ihle-Hansen, S. Ingebrigtsen, C. Kremer, S. B. Krogseth, C. Kruuse, M. Kurz, I. Nakstad, V. Novotny, H. Næss, R. Qazi, M. K. Rezaj, D. M. Rørholt, L. H. Steffensen, J. Sømark, H. Tobro, T. C. Truelsen, L. Wassvik, K. L. Ægidius, D. Atar, Anne Hege Aamodt, NOR-FIB study group

**Affiliations:** 1 Department of Neurology, Østfold Hospital Trust, Grålum, Norway; 2 Institute of Clinical Medicine, University of Oslo, Oslo, Norway; 3 Department of Cardiology, Østfold Hospital Trust, Grålum, Norway; 4 Department for Neurology, Nordlandssykehuset, Bodø, Norway; 5 Department of Neurology, Innlandet Hospital Trust, Lillehammer Hospital, Lillehammer, Norway; 6 Research Institute of Internal Medicine, Oslo University Hospital, Oslo, Norway; 7 Department of Neurology, Molde Hospital, Molde, Norway; 8 Department of Internal Medicine, Diakonhjemmet Hospital, Oslo, Norway; 9 Stroke Unit, Oslo University Hospital, Ullevål, Oslo, Norway; 10 Department of Internal Medicine, Vestre Viken Hospital Trust, Bærum Hospital, Gjettum, Norway; 11 Department of Neurology, University Hospital of North Norway, Tromsø, Norway; 12 Department of Neurology, Skåne University Hospital, Malmö, Sweden; 13 Department of Clinical Sciences, Lund University, Lund, Sweden; 14 Department of Neurology, Vestfold Hospital, Tønsberg, Norway; 15 Department of Neurology, Herlev Gentofte Hospital, Herlev, Denmark; 16 Department of Neurology, Stavanger University Hospital, Stavanger, Norway; 17 Department of Neurology, Vestre Viken Hospital Trust, Drammen Hospital, Drammen, Norway; 18 Department of Neurology, Haukeland University Hospital, Bergen, Norway; 19 Department of Neurology, Telemark Hospital, Skien, Norway; 20 Department of Neurology, Rigshospitalet University Hospital, Copenhagen, Denmark; 21 Department of Neurology, Bispebjerg University Hospital, Copenhagen, Denmark; 22 Department of Cardiology, Oslo University Hospital, Ullevål, Oslo, Norway; 23 Department of Neurology, Oslo University Hospital, Rikshospitalet, Oslo, Norway; 24 Department of Neuromedicine and Movement Science, Norwegian University of Science and Technology (NTNU), Trondheim, Norway

**Keywords:** Cryptogenic stroke, Atrial fibrillation, ICM, Predictors, Prediction scores, Biomarkers

## Abstract

**Background:**

Atrial fibrillation (AF) detection and treatment are key elements to reduce recurrence risk in cryptogenic stroke (CS) with underlying arrhythmia. The purpose of the present study was to assess the predictors of AF in CS and the utility of existing AF-predicting scores in The Nordic Atrial Fibrillation and Stroke (NOR-FIB) Study.

**Method:**

The NOR-FIB study was an international prospective observational multicenter study designed to detect and quantify AF in CS and cryptogenic transient ischaemic attack (TIA) patients monitored by the insertable cardiac monitor (ICM), and to identify AF-predicting biomarkers. The utility of the following AF-predicting scores was tested: AS5F, Brown ESUS-AF, CHA_2_DS_2_-VASc, CHASE-LESS, HATCH, HAVOC, STAF and SURF.

**Results:**

In univariate analyses increasing age, hypertension, left ventricle hypertrophy, dyslipidaemia, antiarrhythmic drugs usage, valvular heart disease, and neuroimaging findings of stroke due to intracranial vessel occlusions and previous ischemic lesions were associated with a higher likelihood of detected AF. In multivariate analysis, age was the only independent predictor of AF. All the AF-predicting scores showed significantly higher score levels for AF than non-AF patients. The STAF and the SURF scores provided the highest sensitivity and negative predictive values, while the AS5F and SURF reached an area under the receiver operating curve (AUC) > 0.7.

**Conclusion:**

Clinical risk scores may guide a personalized evaluation approach in CS patients. Increasing awareness of the usage of available AF-predicting scores may optimize the arrhythmia detection pathway in stroke units.

## Introduction

The potential for diagnosing underlying atrial fibrillation (AF) in cryptogenic stroke (CS) is clinically relevant and may be crucial to the treatment regimen. Current knowledge suggests that one in three CS patients may be diagnosed with AF using prolonged cardiac rhythm monitoring [1, 2]. The duration of monitoring needed to detect paroxysmal arrhythmias seems to be inversely proportional to arrhythmia burden [3], so to properly rule out AF longer monitoring is of interest. Insertable cardiac monitors (ICMs) are the most effective tool revealing AF in up to 58% CS patients [4, 5]. The recent *European Stroke Organisation (ESO) guideline on screening for subclinical atrial fibrillation after stroke or transient ischaemic attack of undetermined origin* recommends ICMs for the purpose of AF detection [6]. However, as most CS patients do not have underlying arrhythmia, the open clinical question is which patients should be prioritized for screening with ICM when the human and economic resources are not unlimited. Therefore, the selection process to pre-clarify individuals at the highest risk for underlying AF, who would profit the most from early ICM usage, is needed. Such an approach will secure high-risk patients’ faster access to this important investigational modality, as well as assuring proper resource utilization in a health-economic perspective. Furthermore, identifying CS patients with the lowest risk for underlying AF may redirect the search for causes other than arrhythmia.

Various risk factors for occult AF have been identified and several predictive scores have been proposed over time, none of them however being widely used in clinical practice [7]. In this paper, we are discussing the significance of AF predictors evaluated in The Nordic Atrial Fibrillation and Stroke (NOR-FIB) Study and the utility of eligible AF-predicting scores in clinical practice.

## Methods

### Study design and outcomes

The NOR-FIB study was an international prospective observational multicenter study designed to detect and quantify the burden of AF (≥ 2 min duration) in patients with CS or cryptogenic transient ischaemic attack (TIA) using an ICM (Reveal LINQ^®^) and to identify biomarkers of incident AF [8]. CS was defined as a non-lacunar brain infarct in the absence of extra- or intracranial atherosclerosis (≥ 50% luminal stenosis in arteries supplying the ischaemic area), high-risk cardiac source (including patent foramen ovale) and any other specific cause of stroke. To avoid bias, clinical TIA cases were included only if an acute lesion on magnetic resonance imaging was detected. The pre-enrolment evaluation and CS diagnosis were assessed by the treating physician. All patients underwent 12-lead ECG and minimum 24-h rhythm monitoring prior to enrolment. Transthoracic echocardiogram was mandatory, while a transesophageal echocardiogram was requested in patients’ ≤ 65 years. Measurements were done according to current guidelines [9–11]. Clinical data on vascular and AF risk factors according to predefined case report forms, and blood samples for biomarkers analyses were collected at enrolment and at a 12-month follow-up visit. All patients were monitored with the ICM for 12 months for arrhythmia detection [12].

Between January 2017 and September 2020, 259 finally included patients with CS or cryptogenic TIA from 18 participating centers in Norway, Denmark, and Sweden were monitored. Systematic ICM data evaluation verified paroxysmal AF or atrial flutter (≥ 2 min duration) in 74 (28.6%) patients. Results regarding arrhythmia detection are previously published [13]. For the purpose of AF prediction, differences between several clinical and paraclinical biomarkers in AF vs non-AF patients and the utility of eight existing AF-predicting scores were evaluated. Blood biomarker analyses were performed separately, showing significantly higher levels of cardiac biomarkers of which N-terminal pro-brain natriuretic peptide (NT-proBNP) was the strongest predictor of underlying AF (OR 4.8 [95% CI 1.8–13.0] in age and sex-adjusted model) [14].

Eligible risk scores tested in the NOR-FIB study, chosen due to data availability for each of the score components were as follows (presented predictive values according to original data*):

- *AS5F score* calculated as Age × 0.76 + National Institutes of Health Stroke Scale (NIHSS) ≤ 5 (9 points) or > 5 (21 points). The threshold of 67.5 points reflects the AF risk of 5.2% (Number Needed to Screen, NNS ≤ 20). Area under the receiver operating characteristic curve (AUC) 0.75. Online calculator available at www.unimedizin-mainz.de/neurologie/header/as5f.html. [15]
- *Brown ESUS-AF score* calculated according to age (65–74 years: 1 point, ≥ 75 years: 2 points) and left atrium (LA) enlargement (moderate or severe: 2 points). Possible total score 0–4. AUC 0.725. For the score of 2 sensitivity 62.9%, specificity 70.6% [16].
- *CHA*_*2*_*DS*_*2*_*-VASc* calculated as 1 point each congestive heart failure, hypertension, diabetes, vascular disease, sex (female) and age (65–74), and 2 points for age ≥ 75 years and prior stroke/TIA. Possible score 0–9. C-index 0.62 [17].
- *CHASE-LESS score* calculated as coronary artery disease (1 point), heart failure (1 point), age (1 point for every 10 years), stroke severity (1 point for NIHSS 6–13, 4 points for ≥ 14), hyperlipidaemia (− 1 point), diabetes (− 1 point), prior stroke/TIA (-1 point). Possible score 1–15. The likelihood of underlying AF categorized as low (1–3), low intermediate (4–6), high intermediate (7–9), high (≥ 10). C-index 0.730 [18].
- *HATCH score* calculated as hypertension (1 point), age > 75 years (1 point), prior stroke/TIA (2 points), chronic obstructive pulmonary disease (1 point), heart failure (2 points). Possible score 0–7. C-index 0.653 [19, 20].
- *HAVOC score* calculated as 1 point each for peripheral vascular disease and obesity (body mass index > 30); 2 points each for hypertension, age ≥ 75 years, valvular heart disease, and coronary disease; 4 points for congestive heart failure. Possible score 0–14. Stratifies patients into three risk groups, low (0–4), medium (5–9) and high (10–14) risk. AUC 0.77. For the score ≥ 4 sensitivity 35%, specificity 82.8%, positive predictive value (PPV) 31.8%, negative predictive value (NPV) 84.7% [21, 22].
- *STAF score* calculated from the sum of the points for the 4 items: age > 62 years (2 points), NIHSS ≥ 8 (1 point), LA dilatation (2 points), absence of symptomatic extra- or intracranial stenosis ≥ 50% or clinic-radiological lacunar syndrome (3 points). Possible total score 0–8. AUC 0.94. The STAF score ≥ 5 identifies patients with AF with a sensitivity of 89% and a specificity of 88% [23].
- *SURF score* calculated as Age × 10 + brain natriuretic peptide (BNP) (ng/l) in the acute phase > 700. CS patients in the NOR-FIB study fulfilled SURF criteria (AF-naive stroke without indication of long-term OAC, no symptomatic atherosclerotic stenosis ≥ 50%, symptomatic arterial dissection or lacunar stroke). AUC 0.842. PPV 47.7%. NPV 96.8% [24].

*The utility of the screening test is usually best expressed by its sensitivity (the percentage of true positive results) and NPV (correctly excluding individuals with no disease), and the AUC or c-index value as the measure of performance.

Cut-off values for the scores were chosen according to the optimal predictive performance from the original papers (AS5F, CHASE-LESS, STAF, SURF), a recommendation from the European Society of Cardiology (Brown ESUS-AF and HAVOC) [25], and comparison between the scores (CHA_2_DS_2_-VASc and HATCH) [15, 26].

### Statistical analysis

Statistical evaluation was performed using IBM SPSS Statistics 28 software. Data were censored at the study exit, time of death or 12-month follow-up. Missing data on risk factors were registered as not present and included in the analysis. Patient characteristics and biomarker levels are presented as frequencies (%), mean (± standard deviation, SD) or median with interquartile range (IQR, Q1–Q3). Independent sample *T*-test or Mann–Whitney *U*-test, according to data distribution, was used to evaluate group differences for continuous variables and Pearson Chi-Square test or Fisher’s exact test for categorical variables. A *p* value < 0.05 was considered significant. Data analyses for arrhythmia detection and blood biomarker assessment are previously described [13, 14]. Binary logistic regression analyses were fitted to estimate the odds ratio (OR) together with a 95% confidence interval (CI). In a multivariate analysis (standard and stepwise), all relevant risk factors were included as covariates. For each patient, the eight AF-predicting scores were calculated and analyzed. The predictive performance of each score was assessed using receiver operating characteristic (ROC) curves, in addition to sensitivity, specificity, PPV and NPV. In the final evaluation, ROC analyses were performed simultaneously for comparison.

## Results

Data from all the 259 were finally included and ICM-monitored patients were evaluated for AF predictors’ detection purpose. Table 1 presents between-group differences in baseline and discharge characteristics indicating potential predictors.

**Table 1:** Characteristics of the NOR-FIB patients

Pres-stroke demographics and comorbidity, *n* = 259	AF group	non-AF group	*p* value
	*n* = 74	*n* = 185	
Age (y), mean (SD)	72.6 (9.7)	62.2 (12.5)	** < 0.001**
Age categories, years *n* (%):			** < 0.001**
< 65	11 (14.9)	98 (53.0)	
65–74	32 (43.2)	56 (30.3)	
≥ 75	31 (41.9)	31 (16.8)	
Female sex, *n* (%)	30 (40.5)	78 (42.2)	0.811
AF risk factors, *n* (%):			
Heart failure^a^	1 (1.4)	2 (1.1)	1
Hypertension^a^	45 (60.8)	87 (47.0)	**0.045**
Diabetes mellitus^a^	6 (8.1)	16 (8.6)	0.888
Prior ischaemic stroke or TIA^a^	18 (24.3)	41 (22.2)	0.708
Haemorrhagic stroke^a^	1 (1.4)	0 (0.0)	0.286
Vascular disease^a^	8 (10.8)	14 (7.6)	0.398
Dyslipidaemia^a^	32 (43.2)	47 (25.4)	**0.005**
Myocardial infarction^a^	4 (5.4)	11 (5.9)	1
Renal failure^a^	5 (6.8)	9 (4.9)	0.55
COPD^a^	5 (6.8)	5 (2.7)	0.155
Cancer^b^	6 (8.1)	11 (5.9)	0.581
Current smoking^c^	8 (10.8)	49 (26.5)	**0.006**
Antiarrhythmic drugs usage^d^	21 (28.4)	23 (12.4)	**0.002**
Echocardiographic findings at enrollment, *n* (%)			
LV hypertrophy, *n* = 234	50 (73.5)	95 (57.2)	**0.020**
Reduced EF^e^, *n* = 242	6 (8.3)	8 (4.7)	0.365
Valvular disease^f^, *n* = 258	30 (40.5)	47 (25.5)	**0.017**
LA enlargement, *n* = 227	28 (45.1)	60 (36.4)	0.251
Mild	10 (16.1)	25 (15.2)	
Moderate	10 (16.1)	13 (7.9)	
Severe	8 (12.9)	22 (13.3)	
Discharge characteristics, *n* (%)			
Heart failure	4 (5.4)	4 (2.2)	0.230
Hypertension	47 (63.5)	98 (53)	0.123
Diabetes mellitus	7 (9.5)	17 (9.2)	0.946
Prior ischaemic stroke or TIA^g^	41 (55.4)	81 (43.8)	0.091
Vascular disease	9 (12.2)	17 (9.2)	0.472
Dyslipidaemia	37 (50.0)	72 (38.9)	0.103
Renal failure	6 (8.1)	13 (7.0)	0.763
CHA_2_DS_2_-VASc median, (IQR)	5 (4–6)	4 (3–5)	** < 0.001**

Bold values mark the significan p value below 0.001

*COPD* chronic obstructive pulmonary disease, *LV* left ventricle, *EF* left ventricular ejection fraction, *LA* left atrium

^a^Self-reported or use of medication at stroke or TIA onset

^b^Previous or current

^c^Current smoking or if stopped < 1 year ago

^d^Including betablockers

^e^EF < 50%

^f^Any type and grade

^g^Self-repported at admission or signs of previous ischaemic lesions on neuroimaging

Summarizing, the AF patients were 10 years older and less often smokers, had higher rates of dyslipidaemia and hypertension pre-stroke and were more often using antiarrhythmic drugs compared to patients without AF. Echocardiographic diagnostic revealed more often left ventricle (LV) hypertrophy and valvular heart disease in AF patients, yet there was no significant difference regarding LA enlargement between the two groups. In addition, index stroke due to intracranial vessel occlusions verified on the angiography (27.4% vs 14.8%, *p* = 0.018) and previous ischaemic lesions on neuroimaging were more often seen in AF patients (51.4% vs 35.7%, *p* = 0.02).

In the univariate analyses, all the above-mentioned potential biomarkers were associated with the risk of AF event detected by ICM. However, in the multivariate analyses only age was associated with an increased likelihood of detecting AF, increasing the odds near 4 times for the age group 65–74 years, and 5 times for patients’ ≥ 75 years old compared to patients aged < 65 years (Table 2). Current smoking was inversely associated with AF risk, which is in line with previously reported findings [7].

**Table 2:** Predictors of underlying AF among patients with CS

Predictor	All	With AF		Univariate OR (95% CI)	Multivariate OR (95% CI)
		*n*	%		
Female	108	30	27.8	Reference	Reference
Male sex	151	44	29.1	1.07 (0.62–1.85)	1.33 (0.68–2.59)
Age, y					
< 65	109	11	10.1	Reference	Reference
65–74	88	32	36.4	5.09 (2.38–10.88)	3.72 (1.55–8.92)
≥ 75	62	31	50.0	8.91 (4.01–19.78)	5.23 (2.0–13.68)
Hypertension^a^					
No	127	29	22.8	Reference	Reference
Yes	132	45	34.1	1.75 (1.01–3.03)	0.71 (0.34–1.50)
Dyslipidaemia^a^					
No	180	42	23.3	Reference	Reference
Yes	79	32	40.5	2.24 (1.27–3.94)	1.45 (0.7–3.02)
Smoking^b^					
No	202	66	32.7	Reference	Reference
Yes	57	8	14.0	0.34 (0.15–0.75)	0.33 (0.12–0.90)
Antiarrhythmic drugs					
No	215	53	24.7	Reference	Reference
Yes	44	21	47.7	2.79 (1.43–5.44)	1.91 (0.81–4.48)
LV hypertrophy					
No	89	18	20.2	Reference	Reference
Yes	145	50	34.5	2.08 (1.02–3.86)	1.86 (0.87–3.97)
Valvular disease^c^					
No	181	44	24.3	Reference	Reference
Yes	77	30	39.0	1.99 (1.12–3.52)	1.0 (0.50–2.01)
Previous ischaemic lesions on neuroimaging					
No	155	36	23.2	Reference	Reference
Yes	104	38	36.5	1.90 (1.10–3.29)	1.47 (0.75–2.86)
Stroke due to intracranial vessel occlusion					
No	209	53	25.4	Reference	Reference
Yes	47	20	42.6	2.18 (1.13–4.21)	1.71 (0.78–3.71)

In the univariate analysis, all admission variables with *p* < 0.05 were included.

In the multivariate analysis, after performed multicollinerity check all variables were included as covariates

^a^Self-reported or use of medication at stroke or TIA onset

^b^Current smoking or if stopped < 1 year ago

^c^Any type and grade

**Fig. 1:**
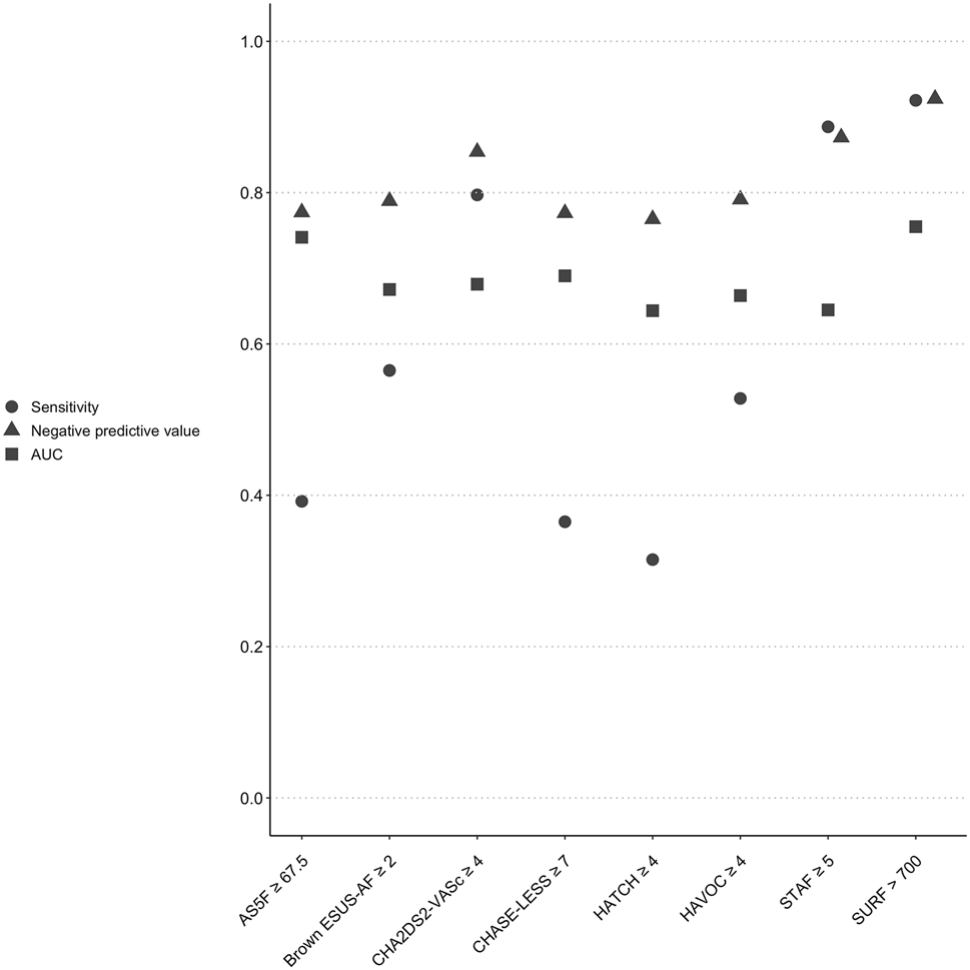
Utility of the eight clinical scores in the NOR-FIB study predicting AF in CS and TIA patients. AUC for continuous score values. Similar AUC results were obtained also when corrected for the n difference between score (all scores tested simultaneously): AS5F 0.719, Brown ESUS-AF 0.674, CHA_2_DS_2_-VASc 0.674, CHASE-LESS 0.664, HATCH 0.639, HAVOC 0.659, STAF 0.673, SURF 0.736

As the second step, we tested the utility of eight eligible clinical risk scores for predicting new-onset AF detected after ischaemic stroke or TIA, performing the analyses on the NOR-FIB dataset (Table 3 and Fig. 1). All the analyses showed significantly higher score levels in AF than non-AF patients (*p* < 0.001) and rates of AF-positive patients due to the increasing score strata (*p* ≤ 0.003). The STAF score and the SURF score provided the highest sensitivities (88.7% and 92.2%) and the highest NPV (87.3% and 92.4%) respectively. AF detection increased with the STAF ≥ 5 to 33.7% vs 12.7% for the score < 5. For the SURF score > 700 AF detection reached 41.8% vs 7.6% for the score < 700. The AUCs based on the continuous score values were as follows: AS5F 0.741 (95% CI 0.678–0.804); Brown ESUS-AF 0.672 (95% CI 0.596–0.747); CHA_2_DS_2_-VASc 0.679 (95% CI 0.606–0.751); CHASE-LESS 0.690 (95% CI 0.619–0.760); HATCH 0.644 (95% CI 0.569–0.719); HAVOC 0.664 (95% CI 0.591–0.736); STAF 0.645 (95% CI 0.566–0.724) and SURF 0.755 (95% CI 0.687–0.824). The AS5F score and the SURF score were the only one considered acceptable reaching AUC > 0.7. AF was detected in 22.6% of patients with the AS5F score < 67.5 and 48.3% of patients with AS5F ≥ 67.5. Performance of the AS5F score was similar compared to validation data, while for the STAF and the SURF score it was lower [15, 26, 27].

**Table 3:** Performance of the eight AF-predicting scores in the NOR-FIB study population

Score type	Sensitivity	Specificity	PPV	NPV	AF vs non-AF patients *p* value
AS5F ≥ 67.5	39.2	83.2	48.3	77.4	< 0.001
Brown ESUS-AF ≥ 2	56.5	64.7	38.9	78.9	0.004
CHA_2_DS_2_-VASc ≥ 4	79.7	47.6	37.8	85.4	< 0.001
CHASE-LESS ≥ 7	36.5	86.5	51.9	77.3	< 0.001
HATCH ≥ 4	31.1	88.1	51.1	76.2	< 0.001
HAVOC ≥ 4	52.8	71.3	42.2	79.1	< 0.001
STAF ≥ 5	88.7	30.8	33.7	87.3	0.003
SURF > 700	92.2	42.7	41.8	92.4	< 0.001

Number of patients for each of the following scores: AS5F: 259, Brown ESUS-AF: 218, CHA_2_DS_2_-VASc: 259, CHASE-LESS: 259, HATCH: 259, HAVOC: 253, STAF: 218, SURF: 207

Cut-off values for the scores chosen according to the optimal predictive performance from the original papers (AS5F, CHASE-LESS, STAF, SURF), recommendation from the European Society of Cardiology [25] (Brown ESUS-AF and HAVOC), and comparison between the scores (CHA_*2*_DS_2_-VASc and HATCH) [15–26].

## Discussion:

The intention of the current analysis, assessing the performance of the eight eligible AF-predicting scores in the NOR-FIB study, was to better address the work-up of identifying high-risk patients to optimize the arrhythmia detection pathway in CS and TIA patients. All the validated clinical prediction scores that were tested; AS5F, Brown ESUS-AF, CHA2DS2-VASc, CHASE-LESS, HATCH, HAVOC, STAF and SURF demonstrated significantly higher score levels in AF than non-AF patients. The STAF and the SURF score predicted AF with the highest sensitivity and NPV, while the AS5F and the SURF score reached an acceptable AUC > 0.7. The SURF score showed however the best utility expressed by the highest levels of all the three values (Fig. 1).

Although AF was common in our study, revealed in nearly 1 in 3 patients systematically monitored by ICM for 12 months, most patients did not have underlying arrhythmia. Our findings emphasize the advantage of risk-stratifying tools for targeted evaluation of CS patients identifying individuals profiting from the early start of prolonged rhythm monitoring. In addition, implementing scores in the diagnostic approach could cover an important but unmet need—the recognition of patients with the lowest risk of underlying AF, in whom the extended evaluation should rather be redirected towards other causes.

### AF predictors in CS

Risk stratification for underlying AF in stroke patients can be attempted by scrutinizing comorbidities predisposing to AF, biomarkers indicating atrial cardiopathy (the substrate for arrhythmia), and neuroimaging lesion patterns indicating embolic stroke [29–33]. Even though increasing age is the most prominent risk factor, burden of comorbidities including hypertension, diabetes mellitus, heart failure, coronary artery disease, chronic kidney disease and obesity significantly contribute to AF development and progression [29]. Several ECG and echocardiographic findings increase the suspicion of atrial disease and AF [34–37]. Elevated cardiac biomarkers levels [14, 38–40] are also reported as a potentially surrogate marker for underlying paroxysmal AF and being incorporated in some of the predictive scores [41, 42]. The latest systematic review and meta-analysis on biomarkers for post-stroke AF detection assessed 69 multimodal markers and identified in all 26 clinical, ECG and blood-based biomarkers [7].

In the NOR-FIB study, several known clinical and paraclinical findings were associated with AF risk in univariate analysis (Table 2). Among the oldest CS patients (≥ 75 years) and those using antiarrhythmic drugs at admission, the likelihood of underlying AF was almost 50%. However, in multivariate analysis, the only independent predictors of AF were increasing age and smoking status. Our findings may be explained by the fact that several of the other AF risk factors are also closely linked, in different overlapping pathologies, to stroke in general. This is further the reason while a single biomarker, including cardiac natriuretic peptides should not be used as a decision-making tool [40], emphasizing the use of scores rather than a stand-alone approach.

### AF-risk stratification scores

Clinical risk scores may guide personalized evaluation approach in CS patients and contribute to more optimal access to key diagnostics. However, the criteria used to define patients at risk of underlying AF should be validated, easy to apply in the first days after admission, and commonly available in all units treating stroke patients. Scores with the highest sensitivity and highest NPV correctly identifying patients at the risk have the best utility. Recent systematic review on the use of risk scores for predicting new AF after stroke or TIA highlights seventeen different scores [41]. Age, LA size, hypertension, congestive heart failure, previous stroke/TIA and NIHSS levels are the throughout most common highly relevant components indicating underlying AF. All the scores are potentially eligible for applying during the diagnostic work-up of stroke patients, yet in more complex scores focus on proper clinical assessment covering the actual score components is essential. Five of these seventeen scores have been developed exclusively for CS or ESUS (embolic stroke of undetermined source) patients to predict AF: AF-ESUS, ACTEL, NDAF, Brown ESUS-AF and HAVOC [17, 18, 44–46]. The first three scores were not applicable to test in the NOR-FIB study due to the use of different variables (LA measurements more often including volume rather than area, lack of specific data on non-stenotic plaques or tricuspid regurgitation). The last two scores, Brown ESUS-AF [16] and HAVOC score [21] showed lower sensitivity and NPV than the STAF and SURF, and lower AUC values than AS5F and SURF scores which predicted AF best. Even though the Brown-ESUS score is similar to the STAF score, the last also includes stroke severity (NIHSS) and may be used in combination with the D-dimer, increasing its accuracy (sensitivity 95%, specificity 100%) [47]. The more comprehensive HAVOC score, however, may be more relevant for younger or multimorbid CS patients’ population. Nevertheless, Brown ESUS-AF and HAVOC scores have lately been proposed by the European Society of Cardiology for the guidance of further prolonged ECG monitoring in CS [25]. In case of negative initial diagnostics (24 h ECG, echocardiography and hematological evaluation), if Brown ESUS-AF ≥ 2 or HAVOC score ≥ 4 prolonged ECG monitoring with external devices up to first 30 days or ICMs directly is recommended. Recently another AF-predicting score for CS patients monitored by ICM has been proposed, PROACTIA score based on premature atrial beats (PAC/24t), P-wave duration, P-wave morphology, and LA end-systolic volume index [48]. The score enables the identification of patients with low, intermediate and a high risk of subsequent AF detection (AUC 0.79), yet has a complicated formula for which the app-based solution is needed (at the moment unavailable) limiting its usability. Another new score stratifying AF risk in CS, the Graz AF Risk Score includes age, NT-proBNP, supraventricular premature beats, atrial runs, atrial enlargement, left ventricular ejection fraction and brain imaging markers [42]. This score was not applicable for us to test either, as we did not count supraventricular premature beats or atrial runs systematically. The usability of both the newest scores may be limited in stroke units mostly applying in-hospital telemetry instead of Holter monitoring, where atrial runs or premature beats counting is less used in the real-time evaluation assessment.

### Proper selection of patients for ICM usage

**Fig. 2:**
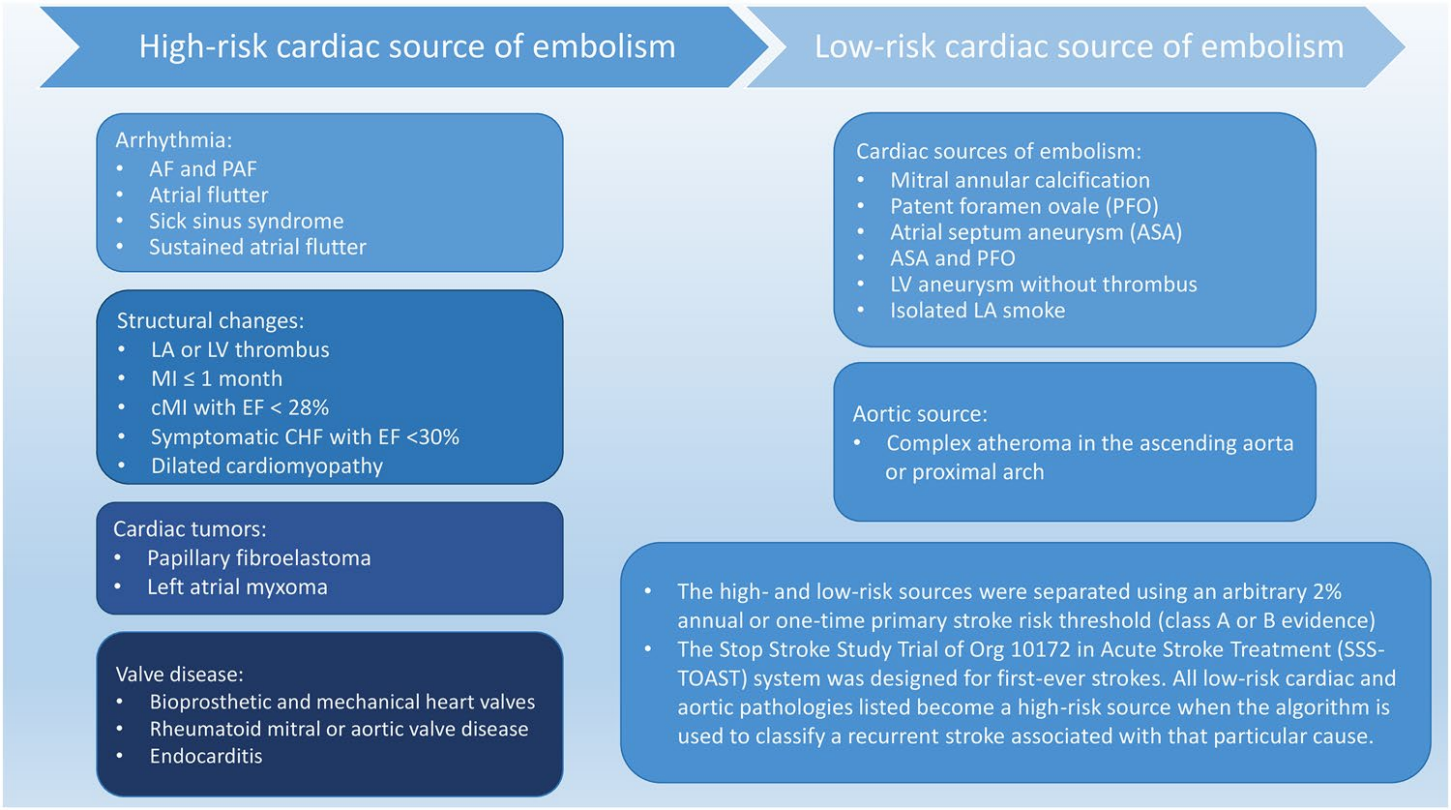
Overview of the cardiac sources of embolism structured after The Stop Stroke TOAST system [49]. AF: atrial fibrillation, PAF: paroxysmal AF, LA: left atrium, LV: left ventricle, MI: myocardial infarction, cMI: chronic myocardial infarction, CHF: congestive heart failure, EF: Left ventricular ejection fraction, TOAST: the Trial of Org 10,172 in Acute Stroke Treatment

Initial work-up of CS requires wide radiological expertise and good quality of the cardiac and vascular evaluation to determine the probable stroke mechanism. Cardiac investigation focusing on the detection of cardio-aortic sources other than AF (Fig. 2), as well as the atrial cardiopathy signs is crucial in diagnosing cardioembolic stroke [49]. Furthermore, clinical or radiological evidence of multiple vascular territory strokes, cortical or posterior circulation lesions, vessel occlusions and greater stroke severity in the absence of significant stenosis in the ipsilateral artery should also lead the suspicion of potentially underlying cardioembolism and AF, if other cardiac sources excluded [33]. In-hospital ECG monitoring for at least 48 h to rule out AF and reflection on other possible causes of brain ischaemia (i.e., genetic disorders and low-risk cardiac sources) should be completed before the stroke may be classified as cryptogenic [6, 50].

For the purpose of prevention, it is realistic to recommend ICM monitoring only to eligible CS patients where underlying AF will affect change in secondary prevention (including those with contraindications to OAC, due to the advent of LA appendage closure procedures). From a socioeconomic perspective, CS patients with short life expectancy (< 1–2 year) or high disability (modified Rankin Scale ≥ 4 due to index stroke or other medical condition with no prospect of recovery), in whom the risk of suffering even a devastating AF-related stroke is less relevant for the outcome should be excluded. Patients with the highest AF suspicion should start prolonged monitoring as early as possible, as recurrent stroke often occurs within the first weeks. The best risk stratification approach is probably using scores combining age (OR 3.26), markers of atrial cardiopathy (OR 2.12–7.79), NIHSS (OR 2.5) and cardiac natriuretic peptides (OR 13.73) which, according to current knowledge, are the strongest predictors of AF (7). The scores that performed best in the present study, the AS5F, STAF and SURF highlight these biomarkers. In a recent study validating AF-predicting scores, the AS5F score demonstrated adequate discrimination (c-index 0.730), being useful in selecting patients for invasive arrhythmia monitoring [27]. The usefulness of the STAF and SURF score is due to its high NPV correctly identifying patients with the lowest AF-risk.

### Limitations

Our study has some minor limitations. First, functional and structural changes in the LA constituting the substrate for AF might have been even more frequent. As completion of the specified case report form for echo data was optional, some of the valuable information on atrial size was missing in 32 patients. Furthermore, the negative impact of hypertension on AF risk might have been leveled by the usage of antihypertensive drugs [43]. At last, there was no formal test of the performance of the evaluated clinical risk scores, however, in simultaneous ROC analysis scores’ AUC were similar to the AUC for continuous variables of each test. The best performance of the STAF, SURF and AS5F score may be due to a high proportion of patients ≥ 60 years (near 70%), yet the proportion ≥ 70 years was 40%, and ≥ 80 years 12%. However, as increasing age has shown to be one of the strongest predictors of AF in several similar studies evaluating biomarkers, and the risk factor that predicted AF among our patients, the findings are not surprising.

## Conclusion

Advanced age and comorbidity scores provided useful information to predict an increased risk of AF in this CS population. Our findings may contribute to a better patient selection for prolonged cardiac rhythm monitoring, by rising awareness on the usage of available AF-predicting scores in optimizing the arrhythmia detection pathway in stroke units. In addition, for patients at the lowest risk of underlying AF the benefit could be an earlier focus on causes other than the arrhythmia. The efforts enhancing the clinical evaluation approach by combining the strongest clinical and paraclinical AF predictors in new scores (or improving the existing) should be prioritized, while new studies and trials should highlight the value of AF prediction as a precision medicine in CS patients.

## Data Availability

The data that support the findings of this study are available, but restrictions apply to the availability of these data, which were used under license for the current study, and so are not publicly available. Data are however available from the authors upon reasonable request, for details please contact Anne Hege Aamodt (a.h.aamodt@medisin.uio.no).
